# Fluoroquinolone heteroresistance in *Mycobacterium tuberculosis:* detection by genotypic and phenotypic assays in experimentally mixed populations

**DOI:** 10.1038/s41598-019-48289-9

**Published:** 2019-08-13

**Authors:** L. Rigouts, P. Miotto, M. Schats, P. Lempens, A. M. Cabibbe, S. Galbiati, V. Lampasona, P. de Rijk, D. M. Cirillo, B. C. de Jong

**Affiliations:** 1 Mycobacteriology Unit, Department of Biomedical Sciences, Institute of Tropical Medicine, Antwerp, Belgium; 2 Department of Biomedical Sciences, University of Antwerp, Antwerp, Belgium; 3 Emerging Bacterial Pathogens Unit, Division of Immunology, Transplantation and Infectious Diseases, IRCCS San Raffaele Scientific Institute, Milan, Italy; 4 Unit of Genomic for the Diagnosis of Human Pathologies, Division of Genetics and Cell Biology, IRCCS San Raffaele Scientific Institute, Milan, Italy

**Keywords:** Infectious-disease diagnostics, Antibiotics

## Abstract

Heteroresistance - the simultaneous presence of drug-susceptible and -resistant organisms - is common in *Mycobacterium tuberculosis*. In this study, we aimed to determine the limit of detection (LOD) of genotypic assays to detect gatifloxacin-resistant mutants in experimentally mixed populations. A fluoroquinolone-susceptible *M. tuberculosis* mother strain (S) and its *in vitro* selected resistant daughter strain harbouring the D94G mutation in *gyrA* (R) were mixed at different ratio’s. Minimum inhibitory concentrations (MICs) against gatifloxacin were determined, while PCR-based techniques included: line probe assays (Genotype MTBDR*sl* and GenoScholar-FQ + KM TB II), Sanger sequencing and targeted deep sequencing. Droplet digital PCR was used as molecular reference method. A breakpoint concentration of 0.25 mg/L allows the phenotypic detection of ≥1% resistant bacilli, whereas at 0.5 mg/L ≥ 5% resistant bacilli are detected. Line probe assays detected ≥5% mutants. Sanger sequencing required the presence of around 15% mutant bacilli to be detected as (hetero) resistant, while targeted deep sequencing detected ≤1% mutants. Deep sequencing and phenotypic testing are the most sensitive methods for detection of fluoroquinolone-resistant minority populations, followed by line probe assays (provided that the mutation is confirmed by a mutation band), while Sanger sequencing proved to be the least sensitive method.

## Introduction

Heteroresistance, a phenomenon defined as co-existence of drug-susceptible (usually wildtype; WT) and drug-resistant (usually mutant) organisms in the same sample or clinical isolate, can result from the concurrent presence of two different strains (mixed infection) or from a changing bacterial subpopulation within the same strain (clonal evolution)^1^. It is common in *Mycobacterium tuberculosis*, has been reported for several antibiotics, and is suggested to cause worse treatment outcome in tuberculosis^2^. Rapid detection of heteroresistance can be important to prevent selection of DR during antibiotics treatment^3^.

The capacity of phenotypic and genotypic assays to detect heteroresistance largely depends on the technique applied. Phenotypic drug-susceptibility testing (pDST), based on the proportion method as originally described by Canetti^4^, aims to determine whether 1% or more of the bacterial population is drug resistant. Accordingly, genotypic assays should be able to detect the simultaneous presence of WT and mutant populations of the relevant genes at different ratios.

Although most studies describing resistance to fluoroquinolones (FQs) among clinical isolates do not provide specific data on heteroresistance, the few studies that do, report frequencies ranging from 14% to 38% of all FQ resistance in some high burden countries, suggesting that FQ specific heteroresistance may be more common than heteroresistance to other anti-TB drugs and relevant for predicting the response to treatment with FQs^2,5–10^. In this study, we aimed to determine the limit of detection (LOD) of genotypic assays to detect gatifloxacin resistant mutants in experimentally mixed populations relative to the LOD of 1% for phenotypic assays. Gatifloxacin is a powerful fourth generation FQ^11^. We compared culture-based determination of the minimum inhibitory concentration (MIC) and PCR-based techniques: line probe assays (LPAs), Sanger sequencing and targeted next generation sequencing (targeted NGS). Droplet digital PCR (ddPCR) served as reference method for absolute quantification and detection of mutant DNA, as its technological principles enable the accurate detection of rare mutations in a background of wildtype sequences, including drug-resistant subpopulations^12–15^.

## Results

**Figure 1:**
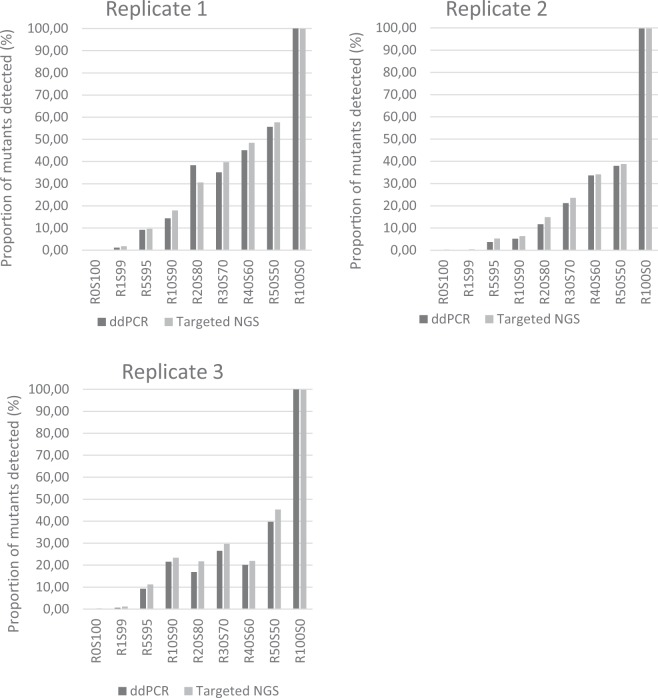
Direct comparison of targeted NGS *gyrA* sequencing with ddPCR for the 3 replicates of experimentally mixed isogenic strains in the following proportions (R%S%): R1S99, R5S95, R10S90, R20S80, R30S70, R40S60, R50S50, R100S0, R0S100.

The average fractional abundance of the D94G allele by ddPCR was ≥99.9% for R100S0 triplicates, while none was detected in R0S100 or H37Rv. In the mixtures, fractional abundance of the D94G mutant was detected with overall increasing frequencies from R1S99 to R50S50, albeit with intra- and inter-experiment variability (Table 1; Fig. 1; See web-only Supplementary Table). In the third experiment, an internal inconsistency was observed with an abundance of 21.6% for R10S90 compared to only 16.9% for R20S80 and 20.1% for R40S60.

**Table 1:** Summary of results from phenotypic and genotypic assays, combined for three triplicates.

Suspension	ddPCR average % (range)	MIC (mg/L)	LPA		Sanger sequence		Targeted NGS average % (range)
			Genotype MTBDR*sl*	GenoScholar-FQ + KM TB II	Software calling	Manual inspection	
**R1**S99	**0.6 (0.42–1.39)**	**0.5**	WT	WT	A	A	**1.13 (0.41–1.82)**
**R5**S95	**7.37 (5.9–10.7)**	**0.5/1.0**	**WT + MUT3C**	WT/**WT + R2c**^a^	A	A	**8.72 (5.31–11.24)**
**R10**S90	**13.73 (12.17–23.3)**	**1.0**	**WT + MUT3C**	WT/**WT + R2c**^a^	A/**R**	A/**R**	**15.9 (6.4–23.35)**
**R20**S80	**18.97 (17.13–30.7)**	**1.0**	**WT + MUT3C**	WT/**WT + R2c**^a^	A/**R**	**R**	**22.39 (14.89–30.56)**
**R30**S70	**27.6 (26.77–37)**	**1.0**	**WT + MUT3C**	**WT + R2c** ^a^	**R**	**R**	**30.95 (23.6–39.64)**
**R40**S60	**32.93 (30.63–47.9)**	**1.0**	**WT + MUT3C**	**WT + R2c**	**R**	**R**	**34.83 (21.91–48.42)**
**R50**S50	**44.43 (42.07–57.8)**	**1.0**	**WT + MUT3C**	**WT + R2c**	**R**	**R**	**47.23 (38.77–57.67)**
**R100**S0	**99.9 (99.78–100)**	**1.0**	**MUT3C**	**R2c**	**G**	**G**	**99.79 (99.73–99.84)**
R0S100	0 (0–0)	<0.25	WT	WT	A	A	0.32 (0.31–0.35)
H37Rv	0 (0–0)	<0.25	WT	WT	A	A	0.27 (0.23–0.35)

Resistance is depicted in bold; ddPCR and NGS data are expressed as fractional abundance of the *gyrA* D94G mutant, with the average and observed range among triplicates; For MIC, LPA and Sanger sequencing the observed results are depict with x/x expressing different results among triplicates; ^a^FQ + KM TB II 2c bands were weakly positive, but also background was observed for the *rrs* mutant bands; *LPA* = *line probe assay*; *ddPCR* = *droplet digital PCR; MIC* = *minimum inhibitory concentration; NGS* = *next generation sequencing*; *WT* = *wildtype*.

Phenotypic analysis showed an MIC < 0.25 mg/L in all triplicates for the fully susceptible control suspension (R0S100) and the H37Rv control strain (Table 1). The R1S99 suspensions had an MIC of 0.5 mg/L in all triplicates (i.e. growth on 0.25 mg/L that exceeded growth on the 1/100 diluted control), whereas the R5S95 suspension showed an MIC of 0.5 mg/L in replicate 2 and 1.0 mg/L in the other two replicates. The higher MIC observed for R5S95 replicates 1 and 3 is in line with the higher than expected ddPCR value of 9.2% mutants for these replicates. The remaining heteroresistant bacterial suspensions and the fully resistant suspension showed an MIC of 1.0 mg/L. Longer incubation of the tubes (three versus six weeks) did not alter MIC values. Applying a breakpoint concentration of 0.25 mg/L would allow detection of ≥1% (R1S99) resistant bacilli, whereas a breakpoint of 0.5 mg/L would allow detecting on average 7.9% (6.5–9.2) (R5S95) resistant bacilli.

On LPA testing, the WT band was absent and the mutant band present for all R100S0 suspensions in both assays, while for the R0S100 and R1S99 suspensions and the H37Rv control strain only the WT band was present. The MTBDR*sl* allowed detection of heteroresistance when on average 7.4% (5.9–8.8)(R5S95) of the mutant bacteria were present, whereas below 3.7% mutants (R1S99) remained undetected (Table 1; See web-only Supplementary Fig. 2). Testing of a single sample set on MTBDR*sl* version 2 did not alter the outcome (See web-only Supplementary Fig. 2). The FQ + KM TB II showed higher variability of the results, with the LOD varying from R5S95 to R30S70 across replicates. The assay was less sensitive, requiring on average ≥17.3% (15.5–19.2) mutants for a clear 2c band to be observed (Table 1; See web-only Supplementary Fig. 3). Testing of the additional sample set with the D94G mutation alone allowed the detection of 5% mutants visualized by probe 2a (See web-only Supplementary Fig. 3).

The automated analysis of Sanger sequences unequivocally revealed the ambiguous ‘R’ call, within the range of 21% to 55% resistant bacilli (Table 1; See web-only Supplementary Fig. 4). Around 15%, sequences showed slightly varying peak heights resulting in different software interpretation: in replicate 1 heteroresistance was detected at 14.4% while in the third replicate 16.9% was scored as WT. Lower proportions systematically showed a WT sequence only (‘a’ nucleotide). Careful manual inspection revealed the presence of additional small peaks distinguishable from background in two cases (11.7% and 16.9%), but not in any of the suspensions with <10% mutants. On average, Sanger sequencing required 15.9% (14.2–17.6) mutant bacteria for detecting the resistant population.

**Figure 2:**
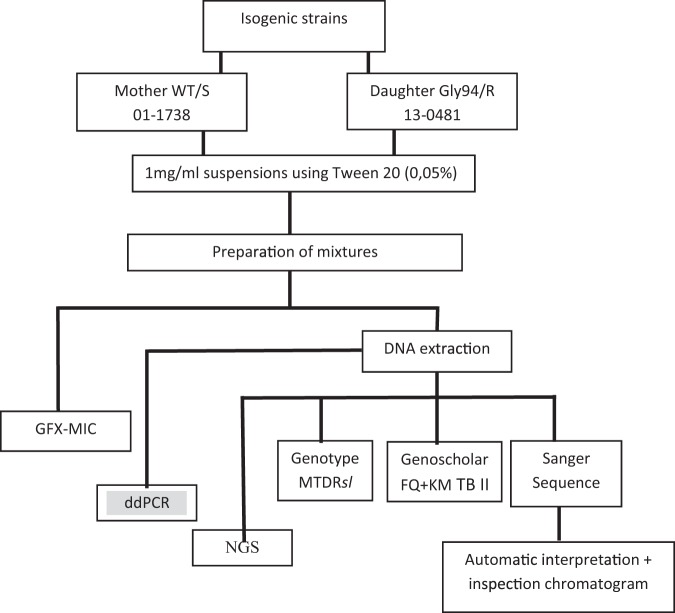
Flowchart on experiment to determine the limit of detection for gatifloxacin heteroresistance. WT = wildtype; S = susceptible; R = resistant; MIC = minimum inhibitory concentration; NGS = next generation sequencing; ddPCR = droplet digital PCR.

Targeted NGS (cluster density: 227 K/mm2; clusters passing filter: 85.5%; ≥Q30: 91.4%; sequence depth >6250 reads) proved to be sensitive enough in detecting heteroresistance in all mixtures (Table 1). Notably, background noise of 0.23% to 0.35% was observed in the R0S100 and H37Rv controls, which may partially explain the overall slightly higher mutant proportions in targeted NGS compared to ddPCR (Table 1; Fig. 2). Nonetheless, observed NGS and ddPCR proportions were tightly correlated.

## Discussion

Overall, pDST and targeted NGS assays achieved the highest sensitivity in detecting gatifloxacin heteroresistance whereas MTBDR*sl* had an LOD around 5% and Sanger sequencing would consistently detect mutants >15%.

While ddPCR results revealed variability among experimentally prepared mixtures used in this study - possibly resulting from pipetting errors or incomplete homogenisation of the mother suspensions - the observed variability among biological triplicate tests was overall within the criteria of acceptability, i.e. for each of the mixtures the intra-triplicate standard deviation was smaller than the intended value to measure.

Recently, the WHO endorsed 0.25 mg/L as critical concentration for gatifloxacin susceptibility testing in MGIT and 0.5 mg/L on Löwenstein-Jensen medium, but no recommendation was given for 7H11 agar medium^16^. Giannoni and colleagues reported all *gyrA* mutants to have a gatifloxacin MIC of ≥0.5 mg/L when tested on 7H11 agar^17^. Our MIC analysis showed that presence of 1 to 3.7% resistant bacilli was associated with an MIC of 0.5 mg/L, while higher mutant proportions resulted systematically in an MIC of 1 mg/L. Hence a breakpoint of 0.25 mg/L on 7H11 would allow to detect approximately 1% D94G mutants, while >5% mutants should be present to be detectable at 0.5 mg/L, favouring a critical concentration of 0.25 mg/L. This observation is consistent with the reported good performance of phenotypic DST to detect heteroresistance^18,19^, including to moxifloxacin^20^.

LPAs are much faster than pDST with results available in just one day when performed directly from a clinical specimen. Direct testing is not only faster, but also avoids selection bias through the culture process, in which one population may out-grow another one^21^. Although it should be considered that visual inspection of LPA strips in our study might have been potentially influenced by the prior knowledge of the involved mutant and expected banding profile, MTBDR*sl* (version 1 and 2) proved slightly less sensitive than phenotypic testing, detecting ~5% D94G mutants, in line with the reported LOD of heteroresistance for rifampicin and isoniazid using MTBDR*plus*^18,19^. Heteroresistance assessment by LPAs is hampered since it is limited to mutants for which a probe is available on the strip, leaving heteroresistance by other mutants undetectable. However, the five mutant probes incorporated in the Genotype MTBDR*sl* assays represent 83% of moxifloxacin resistant strains worldwide (A90V, S91P, D94A/N/Y/G/H)^22^, limiting the impact of this shortcoming. The FQ + KM TB II includes only three of these most prevalent mutations (A90V, D94G/A). In addition, the lower sensitivity of probe 2c decreases the ability to detect heteroresistance in case of combined D94G + S95T polymorphism. Finally, further investigation would be required to ascertain if LPA profiles from direct testing from clinical specimens will result in equally clear bands as those from indirect testing.

As shown previously for rifampicin, isoniazid and moxifloxacin^18–20^, Sanger sequencing was found to be less sensitive in detecting heteroresistance than pDST and LPAs, with the need for the presence of about 15% mutant bacilli to detect heteroresistance using common softwares. On the other hand, sequencing has the advantage that it can detect all genetic variants in the sequence under investigation, and the technique is widely implemented in reference laboratories. Manual re-investigation of the Sanger chromatograms further improved the LOD to 10–15%. Such manual reading is however rather subjective and time consuming.

NGS offers new opportunities to provide comprehensive information for surveillance and clinical management of drug-resistant TB^23–25^. Daum and colleagues identified FQ heteroresistance in 15% of the *M. tuberculosis* isolates by whole genome sequencing (WGS), even with an average reproducible coverage depth of around 30x^26^. Deep targeted sequencing generates more copies per position compared to standard WGS, increasing the probability of detecting minority populations^9,27^. Our current findings confirm that ultra-deep sequencing (>5000x) is suitable to detect as little as 1% of resistant bacteria in a mixed population. However, since our study was limited to the analysis of just D94G, it remains to be proven that NGS would have equivalent performance for other mutants.

In general, all approaches tested provided more accurate estimates for FQ heteroresistance compared to melting temperature assays using molecular beacons or dual-labelled probes, previously reported to require for detection of heteroresistance 10–70% and 30–100% of mutants, respectively^28^. GeneXpert MTB/RIF Ultra – using melting curves to detect rifampicin resistance – can detect 5–40% mutants depending on the mutation type^29^.

The clinical importance of heteroresistance is likely substantial, akin to’fixed‘ (100%) resistance. The proportion method for pDST, which has been around for over half a century, by design, tests for ≥1% resistant subpopulations, with strong predictive value for poor treatment outcome, at least for the core drugs like FQs and rifampicin^30^. High-level FQ resistance has been associated with poor treatment outcome^31,32^, and specific *gyrA* mutations like D94G predict poor outcome^32^. In the same study, software based Sanger sequencing detected heteroresistance in 9.2% of *gyrA/B* mutants^32^. While the sample size was too small to identify treatment outcome differences between heterogeneous versus homogenous populations, their MIC levels fell in the same range, which is in agreement with the current MIC findings. Taken together, this implicates that any (molecular) detection of ≥1% heteroresistance, is probably key (for core drugs), notwithstanding the lack of direct evidence on clinical impact.

In conclusion, targeted NGS analysis reached an LOD of at least 1% mutant variants, equalling the 1% critical concentration applied for pDST, whereas LPAs and Sanger sequencing required higher mutant proportions to be detectable (5% and 15% respectively). Their direct application on (heteroresistant) clinical samples requires further evaluation. Based on indirect testing, our findings suggest that targeted deep sequencing may become the gold standard for the detection of heteroresistance directly in clinical samples. The LOD of direct testing, including on samples with low bacterial burden such as Xpert-positive microscopy-negative samples, will need to be determined in future studies, including the clinical relevance of small proportions of mutant populations.

## Methods

### Experimentally mixed populations

FQ resistance in *M. tuberculosis* is mainly caused by mutations in the FQ-resistance determining regions in the *gyrA* and *gyrB* genes, encoding for DNA gyrase, the target of FQs^22^. Two isogenic strains were used to minimize bias by inter-strain variability: a pan-susceptible mother strain with a phylogenetic polymorphism in *gyrA* (ITM 011738, S; *gyrA* agc95acc (S95T) and *gyrB* WT) and it’s *in vitro* selected ofloxacin-resistant daughter showing in addition a *gyrA* gac94ggc D94G mutation (ITM 130481) (see web-only See web-only Supplementary M&M). D94G was chosen as it represents 21–32% of FQ-associated mutations in clinical isolates and has been related to high MICs, negatively impacting MDR-TB treatment outcome^32^. For each strain, a 1 mg/mL bacterial suspension was prepared by transferring colonies from 3-weeks old Löwenstein-Jensen slants in sterile 0.01% Tween80 solution, followed by 1 minute vortexing, as per laboratory’s routine practice. Homogeneous suspensions were mixed in the following proportions (R%S%): R0S100, R1S99, R5S95, R10S90, R20S80, R30S70, R40S60, R50S50, R100S0. These suspensions were prepared thrice on different days to generate biological triplicates allowing to determine reproducibility of results (Fig. 2). The H37Rv *M. tuberculosis* reference strain (BCCM/ITM 083715) was included as control in each experiment.

To check the mutant/WT proportion in each of the mixtures and replicates, droplet ddPCR analysis was performed on a QX100 ddPCR system (Bio-Rad, Hercules, CA) using PrimePCR Custom Assay hydrolysis probes (See web-only Supplementary M&M; See web-only Supplementary Fig. 1).

### MIC determination

The MIC for gatifloxacin was determined on Middlebrook 7H11 agar medium at 0.25, 0.5, 1.0, 2.0 and 4.0 mg/L after three and six weeks of incubation as described before^32^. A difference of 1 dilution in MIC values among triplicates was considered acceptable.

### Heat inactivation

DNA extraction by heat inactivation was performed as previously described^32^. Heat inactivated suspensions were shipped by courier at ambient temperature from Antwerp (Belgium) to Milan (Italy) for targeted NGS and ddPCR.

### Sanger sequencing of the *gyrA* and *gyrB* genes

**Figure 3:**
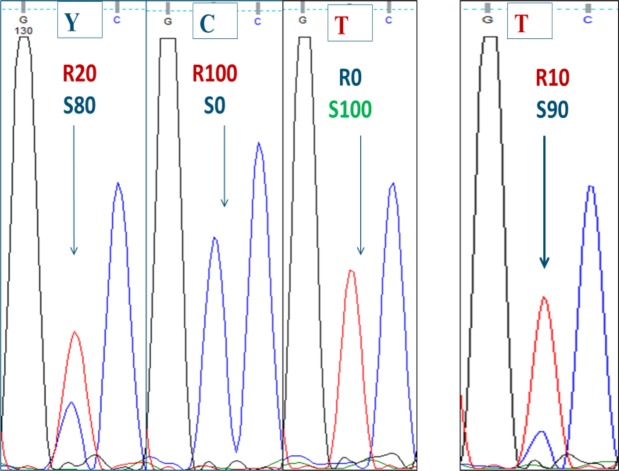
Examples of Sanger sequence analysis from the *gyrA* gene for experimentally mixed strains, representing the reverse string. Left: automated software interpretation (limit of detection for FQ heteroresistance = 20%) Right: manual interpretation showing a double peak that remained undetected by the software (limit of detection = 10%). *R10S90* = *10% mutant bacilli* + *90% susceptible bacilli; R20S80* = *20% mutant bacilli* + *80% susceptible bacilli; RS100* = *100% susceptible bacilli; R100S0* = *100% mutant bacilli*.

Amplification of *gyrA* and *gyrB* was done as described^33^. An isolate was defined as heteroresistant if both WT nucleotides and mutant nucleotides conferring resistance at a specific locus were detected, either by the CLC Sequence Viewer software (version 7.0.1) calling ‘ambiguous’ nucleotides like ‘R’ representing the simultaneous detection of a and t nucleotides, or by blinded manual re-inspection of the chromatograms using Finch TV (version 1.4.0) (Geospiza Inc.) (Fig. 3). For the manual interpretation, we only considered peaks that were centered under the main peak and of which the height exceeded background noise (See web-only Supplementary M&M).

### GenoType MTBDR*sl* (version 1, Hain Lifescience, Germany) and GenoScholar-FQ + KM TB II (Nipro, Japan)

**Figure 4:**
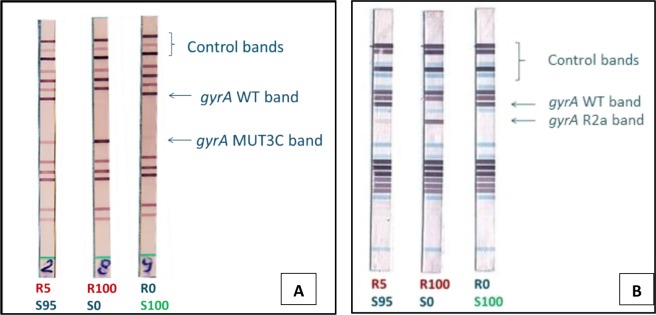
Examples of MTBDR*sl* (V1) (**A**) and Genoscholar FQ + KM TB II (**B**) strips from experimentally mixed strains, showing a limit of detection of 5% resistant bacilli (WT + MUT band present). The Genoscholar FQ + KM TB II strips show the results of the mixtures with an isolate containing the D94G mutant, yet not having the S95T polymorphism. *WT* = *wildtype; MUT* = *mutant; R5S95* = *5% mutant bacilli* + *95% susceptible bacilli; RS100* = *100% susceptible bacilli; R100S0* = *100% mutant bacilli*.

DNA amplification and hybridization were done according to the manufacturers’ instructions using the Twincubator (MTBDR*sl*) or Multiblot NS-4800 (FQ + KM TB II providing a fully automated hybridization process). To document non-inferiority of the newly released version 2 of the MTBDR*sl*, one set of samples was repeated. The LPA profile readout was performed manually by two readers. In case of heteroresistance with a D94G mutation, the MUT3C band in MTBDR*sl* and all WT bands of the *gyrA* gene had to be visible, whereas complete resistance would result in the absence of WT3 in presence of MUT3C, and complete susceptibility in the absence of MUT3C in presence of all WT bands (Fig. 4A). Similar profiles would be obtained for the FQ + KM TB II, yet with the 2c band reacting, representing the combined D94G and S95T polymorphism (Fig. 4B). To challenge the 2a probe – representing the D94G mutation alone – another set of mixtures with a WT strain (H37Rv) and a D94G mutant (ITM 102197) was tested once with the FQ + KM TB II.

### Illumina targeted NGS

A fraction of the *gyrA* gene including the QRDR (genomic coordinates: 7077-7721; amplicon length: 645 bp) was amplified using a standard amplification protocol (See web-only Supplementary M&M).

## Supplementary information

Supplementary info

## Data Availability

Upon request, materials or information described in this study can be made available without restrictions, apart from the experimental mixtures that have been used almost entirely for these and additional analysis.
