# Supplementary material for: Fluoroquinolone heteroresistance in Mycobacterium tuberculosis: detection by genotypic and phenotypic assays in experimentally mixed populations
Source: Sci Rep. 2019 Aug 13;9:11760. doi: 10.1038/s41598-019-48289-9 (PMC6692311; doi:10.1038/s41598-019-48289-9)
Supplement: Supplementary file 1 — Supplementary info [file 41598_2019_48289_MOESM1_ESM.pdf]

Fluoroquinolone heteroresistance in *Mycobacterium tuberculosis:* detection by genotypic and phenotypic assays in experimentally mixed populations.

Rigouts L, Miotto P, Schats M, Lempens P, Cabibbe AM, Galbiati S, Lampasona V, de Rijk P, Cirillo DM, de Jong BC

SUPPLEMENTARY DATA MATERIALS AND METHODS

Induction of FQ-resistant daughter strain.

FQ resistance was induced by growing the mother strain in Middlebrook 7H9 broth enriched with OADC, glycerol and 0.05% Tween80, and inoculating one drop of the 4-weeks old culture on a 7H10 agar plate containing 1 mg/L ofloxacin. A single colony from the plate was subcultured on plain Löwenstein-Jensen medium, and this daughter strain had MIC for gatifloxacin determined.

ddPCR and Illumina targeted NGS

To check the mutant/wildtype proportion in each of the mixtures and replicates, droplet digital PCR (ddPCR) analysis was performed on a QX100 ddPCR system (Bio-Rad, Hercules, CA) using PrimePCR Custom Assay hydrolysis probes.

Primers and probes for *gyrA* were designed based on the sequences of the isogenic strains (Table). The assay was optimized using previously characterized genomic DNA samples from clinical isolates showing the following profiles in *gyrA* by Sanger sequencing: D94G, S95T, WT+D94G, WT+D94G+S95T, WT (see Figure 1 manuscript). Fluorometric quantitation of DNA was performed on all tested samples by the Qubit dsDNA HS kit (Thermo Fisher Scientific) according to manufacturer's instructions. DNA from each mixture was diluted (1:1000) in nuclease free water and subjected to ddPCR in order to establish the correct amount of DNA to be tested. PCR reactions were prepared with ddPCR Supermix (no-dUTPs) (Bio-Rad), primers and WT and mutant probe, 2 µL of a 1:1000 dilution of DNA template and water up to 20 µL and partitioned by a QX100 droplet generator according to the manufacturer's instructions. 40 µL of emulsified PCR reactions were run on a 96-well plate on a T100 Bio-Rad thermal cycler, incubating the plates at 95 °C for 10 min, followed by 40 cycles of 94 °C for 30 s and 55 °C for 60 s, then by a 10 min incubation at 98 °C. The temperature ramp increment was 2 °C/s for all steps. Plates were read on a Bio-Rad QX100 droplet reader using the QuantaSoft version 1.7.4.0917 software from Bio-Rad. At least two negative control wells with no DNA template were included in each run.

For Illumina targeted sequencing, a fraction of the *gyrA* gene including the QRDR (genomic coordinates: 7077-7721; amplicon length: 645 bp) was amplified using a standard amplification protocol. Briefly, each amplification reaction contained 1.5 mM $MgCl_2$, 0.2 mM of each deoxynucleoside triphosphate, 20 pmol of each primer, 2.5 U of HotStartTaq DNA Polymerase (Qiagen), and 5 µL of lysate in 50 µL of molecular grade water.

Amplification was performed in an iCycler thermal cycler (Bio-Rad) with an initial cycle at 95 °C for 15 min (Taq polymerase activation step), followed by 40 cycles at 95 °C for 1 min, 59.5 °C for 1 min, and 72 °C for 1 min, and final extension at 72 °C for 8 min. Amplicons were purified by standard sodium acetate/ethanol precipitation and checked by gel electrophoresis. Details on the primers can be found in Table.

Amplicons were quantified in a Qubit 2.0 Fluorometer (Thermo Fisher Scientific) using the Qubit dsDNA HS kit according to manufacturer's instructions. Library preparation was performed according to the Nextera XT DNA Library Preparation kit (Illumina Inc.) for preparation of 30 indexed paired-end libraries. Library normalization and pooling were carried out prior to run the samples on a MiniSeq sequencer (Illumina Inc.). The sequencing results were processed for demultiplexing, reads quality filtering (MinQScore: 20), alignment to the H37Rv reference genome and calling variants at the genomic position 7582 (a→g, D94G) of interest.

Table: Primer and probe sequences used for ddPCR and targeted NGS.

Applied technique	Primer name	Sequence	Dye	Mapping position (on +strand)
ddPCR	Forward	3’-CAACTACCACCCGCA-5’	none	7548-7562
ddPCR	Reverse	3’-ACCAGGGCTGGGC-5’	none	7600-7612
ddPCR	WT probe	3’-TCTACG**A**CACCCTGGT-5’	Dye Quencher: 5’ HEX, 3’lowa Black FQ	7577-7592
ddPCR	MUT probe	3’-TCTACG**G**CACCCTGG-5’	5’ 6-FAM, 3’lowa Black FQ	7577-7592
NGS	gyrA_Illumina_f	5’-TAGGTGAAATGGACGCTAAGG-3’	None	
NGS	gyrA REV	5’-CTCCATCGCCAACGGG-3’	None	

Nucleotides in bold represent the gac94ggc D94G substitution; Underlined nucleotides represent the agc95acc S95T polymorphism.

Sanger sequencing:

Amplification of *gyrA* and *gyrB* was done in Antwerp (Belgium) as described.[19] CLC Sequence Viewer (version 7.0.1) was used to identified by comparing the results to the *M. tuberculosis* H37Rv reference sequence (GenBank accession no. NC_000962.2) using the NCBI BLAST server.

Examples of chromatograms and the interpretation in case of (potential) double peaks: **A**: ambiguous nucleotide called by the CLC software is considered as true heteroresistance; **B:** manually observed double peaks not called as ambiguous by the CLC software, yet considered as possible heteroresistance given the central position and the height of the smallest peak compared to background noise; **C:** manually observed 'double peaks' not called as ambiguous by the CLC software, and not considered as heteroresistance given the decentral position and/or marginally higher top compared to surrounding background noise.

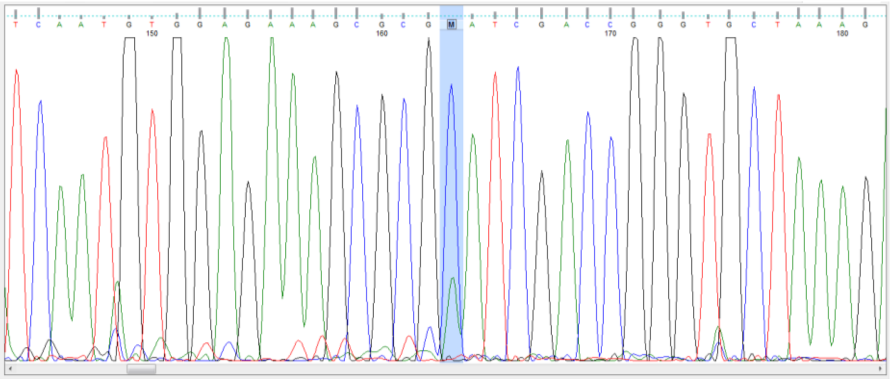

A

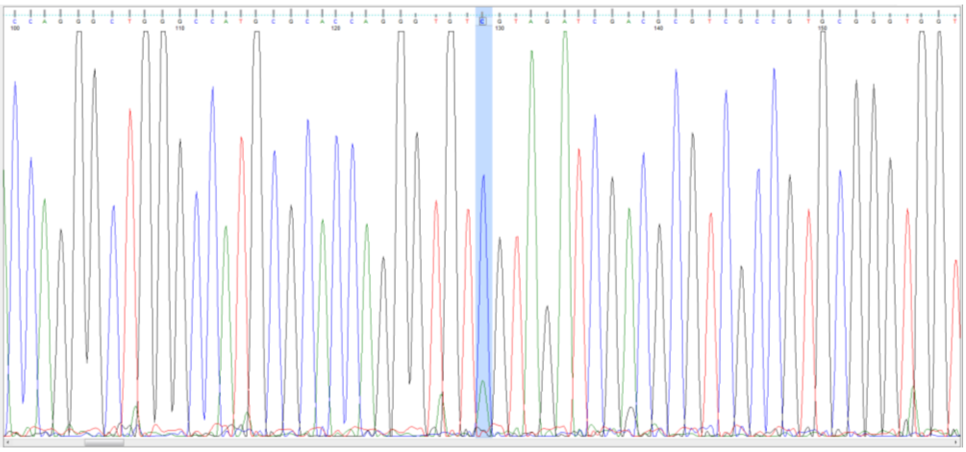

B

C

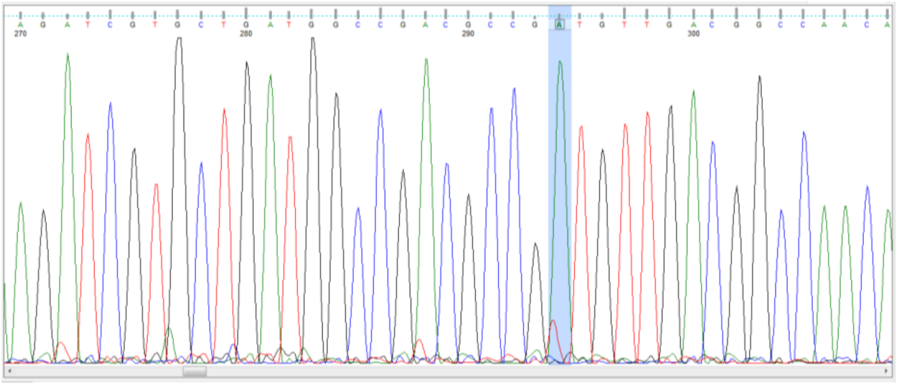
A G A T C G T G C T G A T G G C C G A C G C C G A T G T T G A C G G C C A A C A
270
280
290
300

SUPPLEMENTARY DATA RESULTS

Supplementary Figure 1 Examples of ddPCR from experimentally mixed strains, showing a limit of detection of 1*% resistant bacilli; R1S99 = 1% mutant bacilli + 99% susceptible bacilli; RS100 = 100% susceptible bacilli; R100S0 = 100% mutant bacilli.*

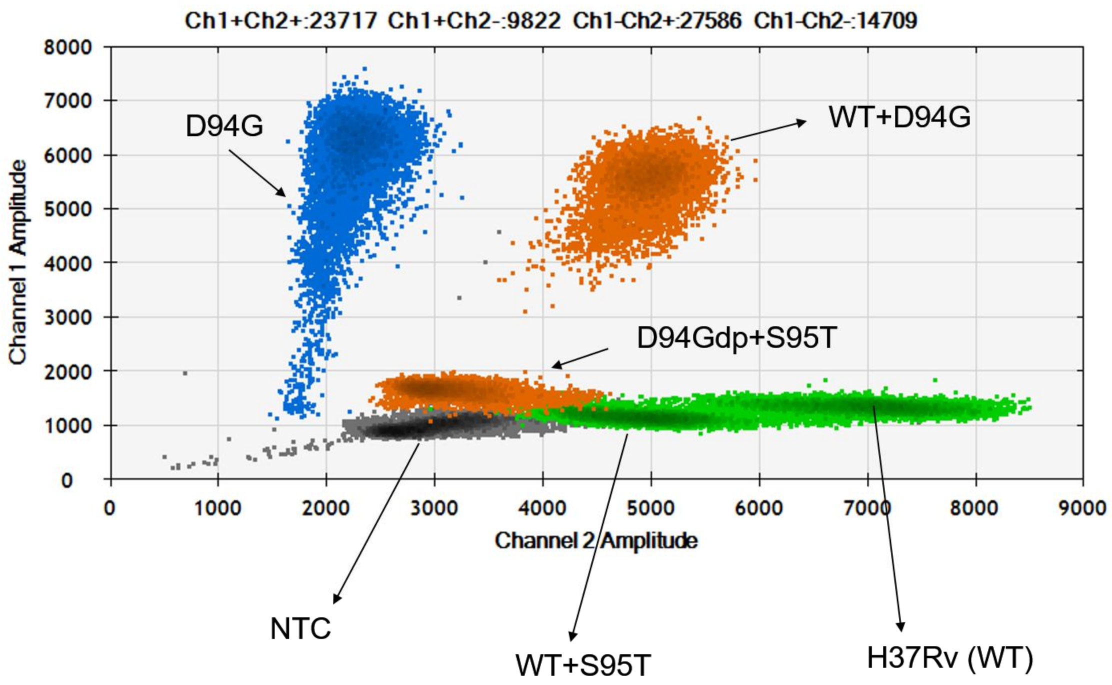

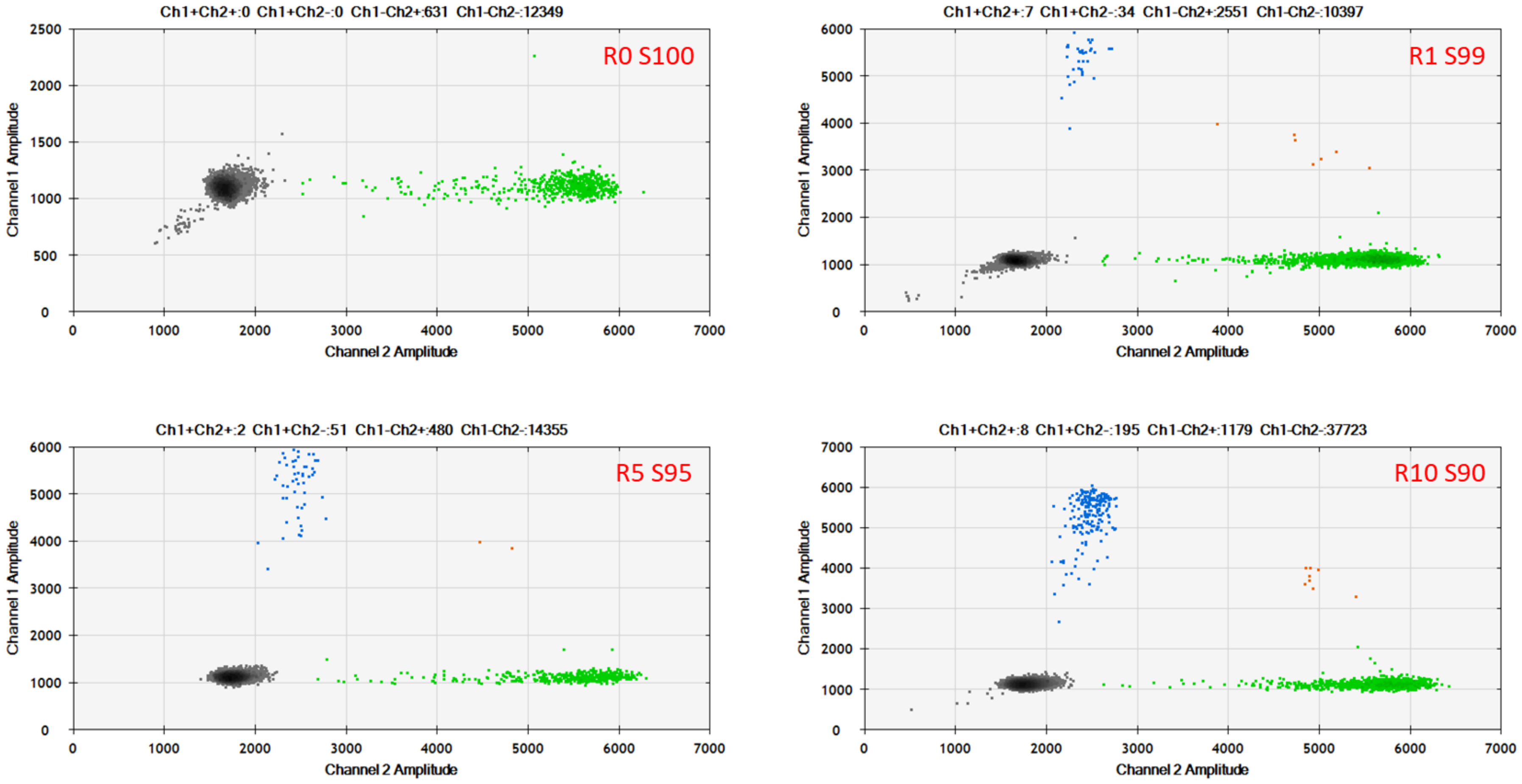
Ch1+Ch2+:0 Ch1+Ch2-:0 Ch1-Ch2+:631 Ch1-Ch2-:12349
R0 S100
Channel 1 Amplitude
Channel 2 Amplitude
Ch1+Ch2+:7 Ch1+Ch2-:34 Ch1-Ch2+:2551 Ch1-Ch2-:10397
R1 S99
Channel 1 Amplitude
Channel 2 Amplitude
Ch1+Ch2+:2 Ch1+Ch2-:51 Ch1-Ch2+:480 Ch1-Ch2-:14355
R5 S95
Channel 1 Amplitude
Channel 2 Amplitude
Ch1+Ch2+:8 Ch1+Ch2-:195 Ch1-Ch2+:1179 Ch1-Ch2-:37723
R10 S90
Channel 1 Amplitude
Channel 2 Amplitude

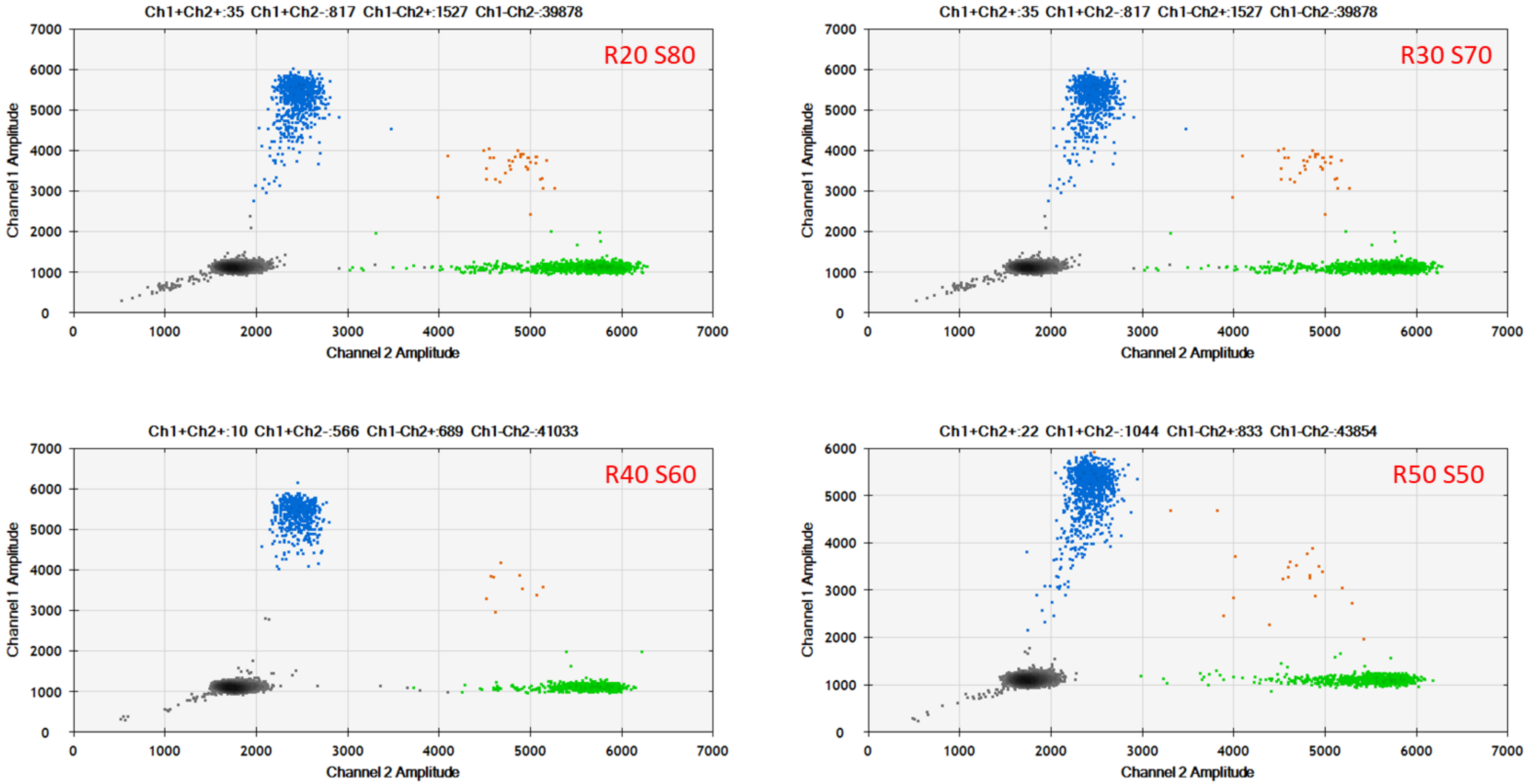

Ch1+Ch2+:35 Ch1+Ch2-:817 Ch1-Ch2+:1527 Ch1-Ch2-:39878
R20 S80
Channel 1 Amplitude
Channel 2 Amplitude
Ch1+Ch2+:35 Ch1+Ch2-:817 Ch1-Ch2+:1527 Ch1-Ch2-:39878
R30 S70
Channel 1 Amplitude
Channel 2 Amplitude
Ch1+Ch2+:10 Ch1+Ch2-:566 Ch1-Ch2+:689 Ch1-Ch2-:41033
R40 S60
Channel 1 Amplitude
Channel 2 Amplitude
Ch1+Ch2+:22 Ch1+Ch2-:1044 Ch1-Ch2+:833 Ch1-Ch2-:43854
R50 S50
Channel 1 Amplitude
Channel 2 Amplitude

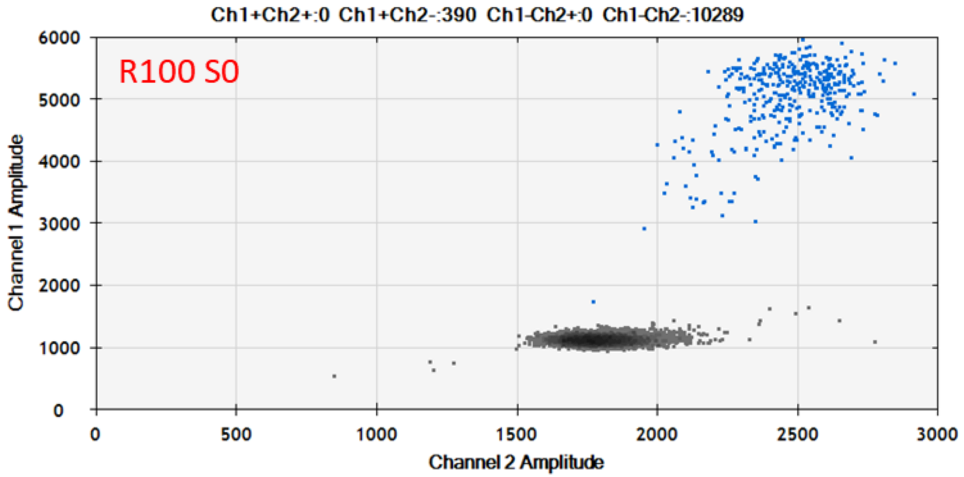
Ch1+Ch2+:0 Ch1+Ch2-:390 Ch1-Ch2+:0 Ch1-Ch2-:10289
R100 S0
Channel 1 Amplitude
Channel 2 Amplitude
0
1000
2000
3000
4000
5000
6000
0
500
1000
1500
2000
2500
3000

Supplementary Figure 2: Photographs of the LPA strips obtained by GenoType MTBDRsl (Hain Lifescience, Germany) of triplicates from experimentally mixed isogenic *Mycobacterium tuberculosis* strains in the following proportions (R%S%): R1S99, R5S95, R10S90, R20S80, R30S70, R40S60, R50S50, R100S0, R0S100, and the H37Rv reference strain (lanes A to J respectively).

More detailed information on the mutant proportion as determined by droplet digital PCR can be found in Table 1 of the manuscript.

All replicates were tested using version 1 of the assay. In addition, replicate 1 was tested with version 2 as well.

Replicate 1 - GenoType MTBDRsl_V1

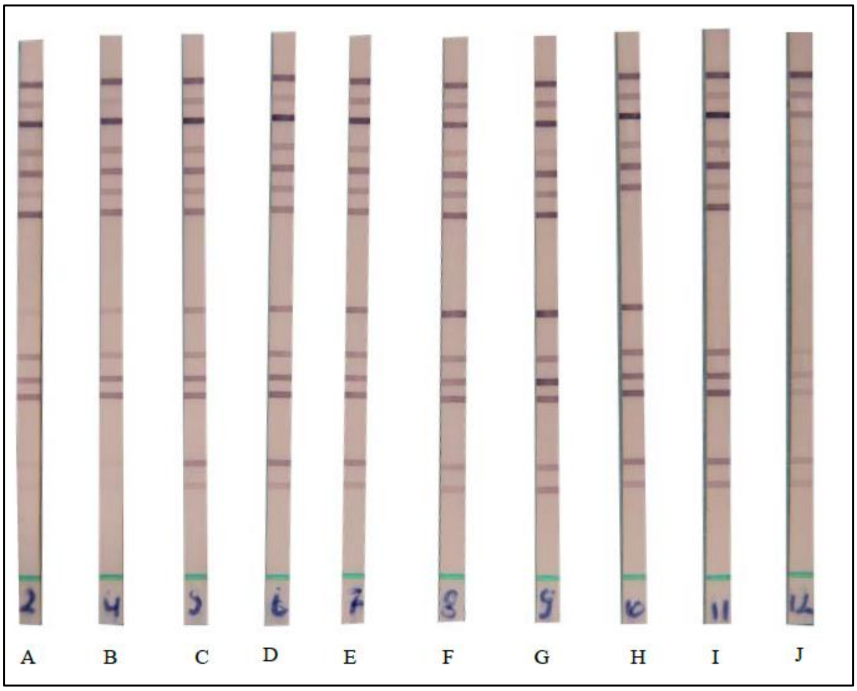

Replicate 2 GenoType MTBDRsl_V1

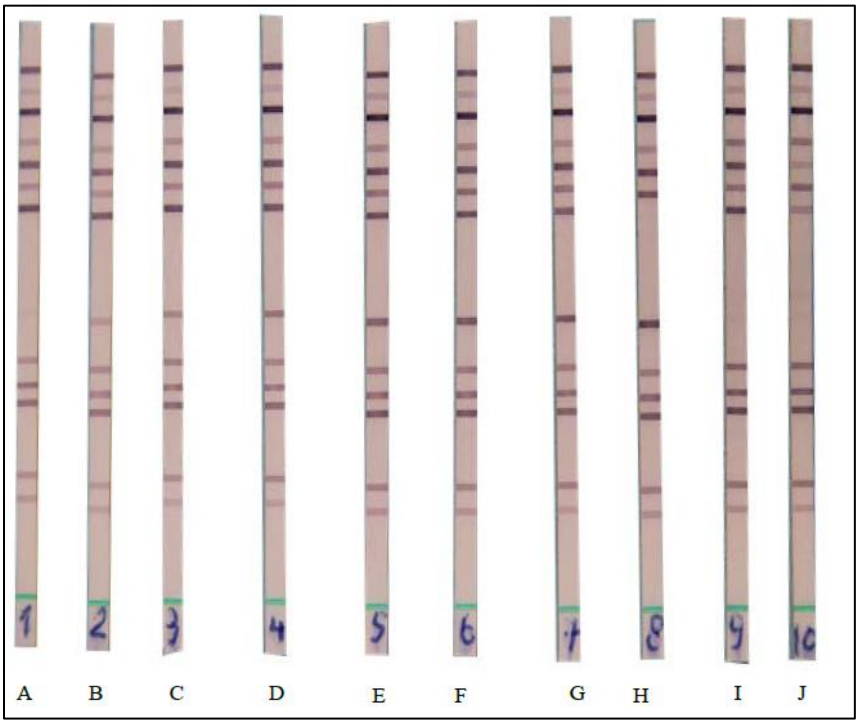

Replicate 3 GenoType MTBDRsl_V1

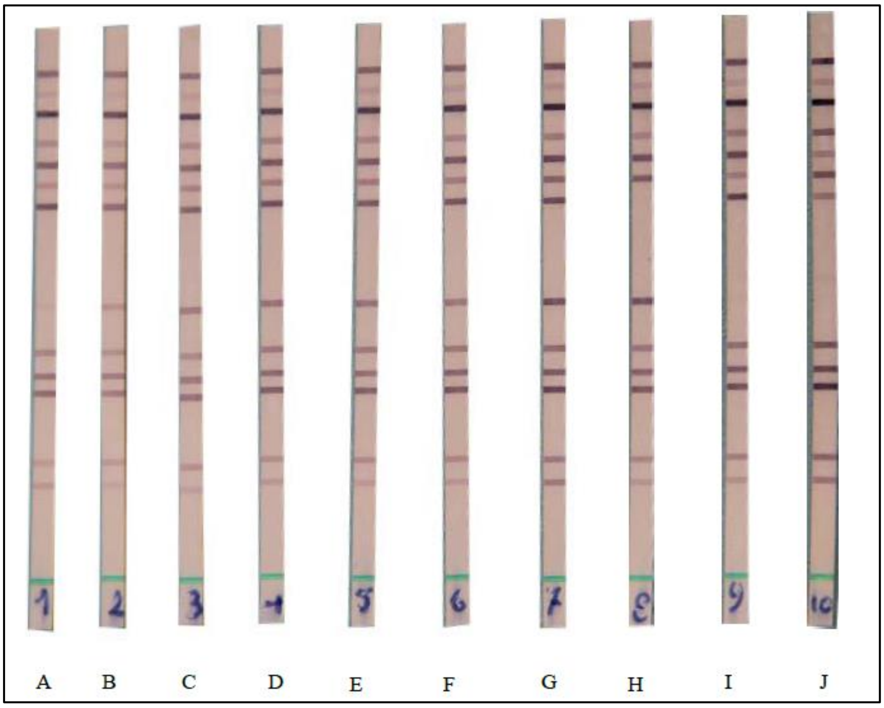

Replicate 1 GenoType MTBDRsl_V2

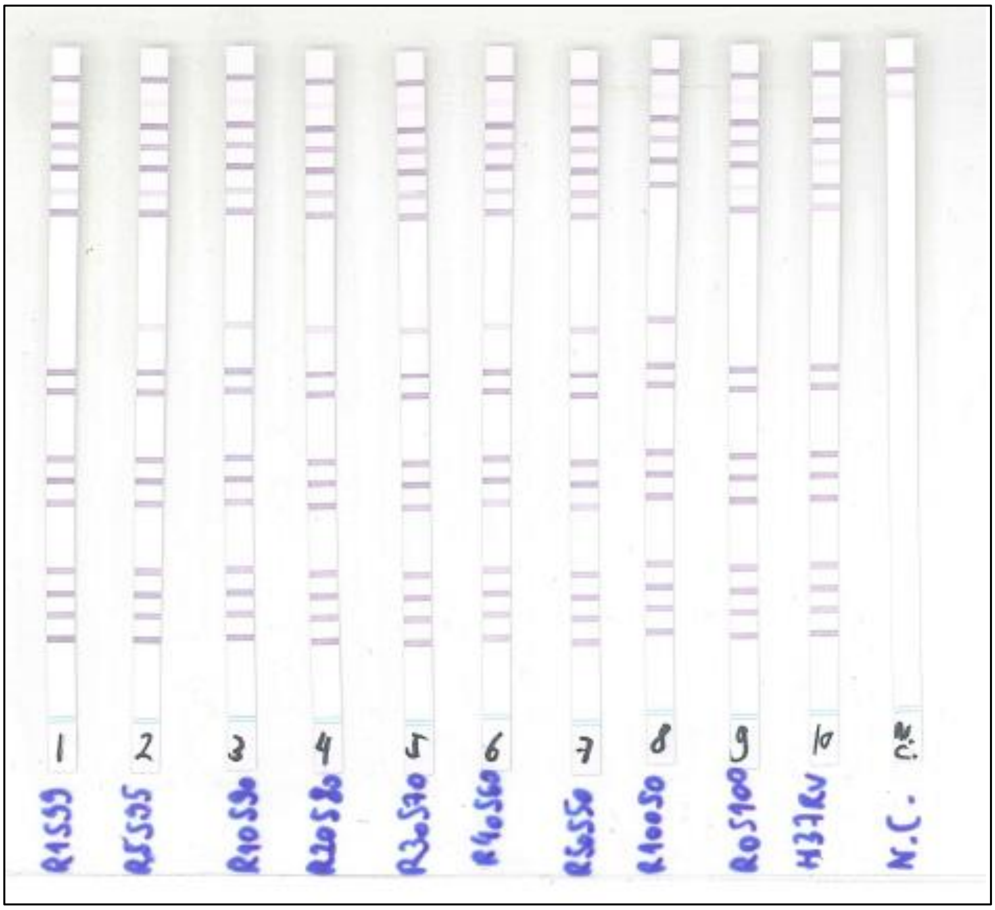

Supplementary Figure 3: Photographs of the LPA strips obtained by Genoscholar FQ+KM TB II (Nipro,Japan) of triplicates from experimentally mixed isogenic *Mycobacterium tuberculosis* strains in the following proportions (R%S%): R1S99, R5S95, R10S90, R20S80, R30S70, R40S60, R50S50, R100S0, R0S100, and the H37Rv reference strain (lanes A to J respectively).

More detailed information on the mutant proportion as determined by digital PCR can be found in Table 1 and the Supplementary Table of the manuscript.

Replicates 1 – 2 - 3 - Genoscholar FQ+KM TB II

Sample	Mix	P-number ITM	Date	Strip
1	R1 S99	p143254	2014/11/13	
2	R5 S95	p143255	2014/11/13	
3	R10 S90	p143256	2014/11/13	
4	R20 S80	p143257	2014/11/13	
5	R30 S70	p143258	2014/11/13	
6	R40 S60	p143259	2014/11/13	
7	R50 S50	p143260	2014/11/13	
8	R100 S0	p143261	2014/11/13	
9	R0 S100	p143262	2014/11/13	
10	H37Rv	p143263	2014/11/13	
11	R1 S99	p150015	2014/12/16	
12	R5 S95	p150016	2014/12/16	
13	R10 S90	p150017	2014/12/16	
14	R20 S80	p150018	2014/12/16	
15	R30 S70	p150019	2014/12/16	
16	R40 S60	p150020	2014/12/16	
17	R50 S50	p150021	2014/12/16	
18	R100 S0	p150022	2014/12/16	
19	R0 S100	p150023	2014/12/16	
20	H37Rv	p150024	2014/12/16	
21	R1 S99	p150130	2015/1/7	
22	R5 S95	p150131	2015/1/7	
23	R10 S90	p150132	2015/1/7	
24	R20 S80	p150133	2015/1/7	
25	R30 S70	p150134	2015/1/7	
26	R40 S60	p150135	2015/1/7	
27	R50 S50	p150136	2015/1/7	
28	R100 S0	p150137	2015/1/7	
29	R0 S100	p150138	2015/1/7	
30	H37Rv	p150139	2015/1/7	
blank	-	-	-	

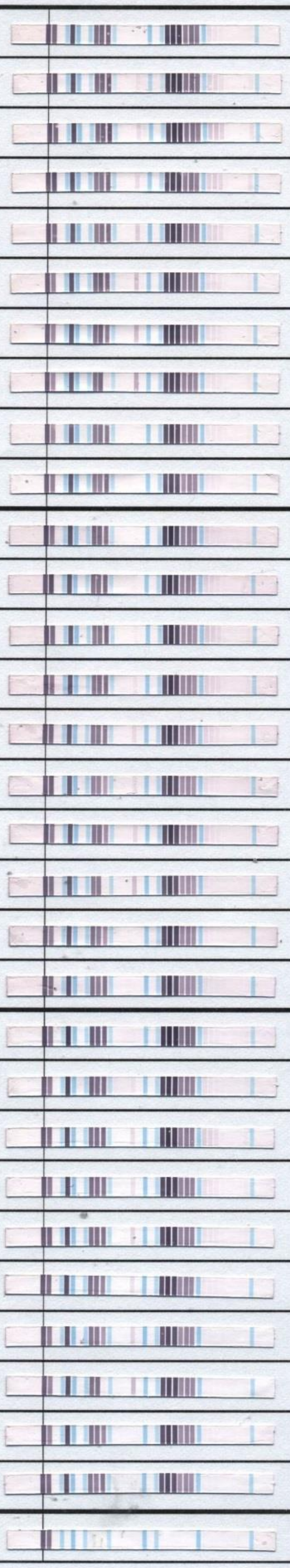

To challenge the 2A probe – representing the D94G mutation alone – another set of mixtures with a WT strain (H37Rv) and a D94G mutant (ITM102197) was tested once with the FQ+KM TB II.

Extra data on additional set of mixtures - Genoscholar FQ+KM TB II

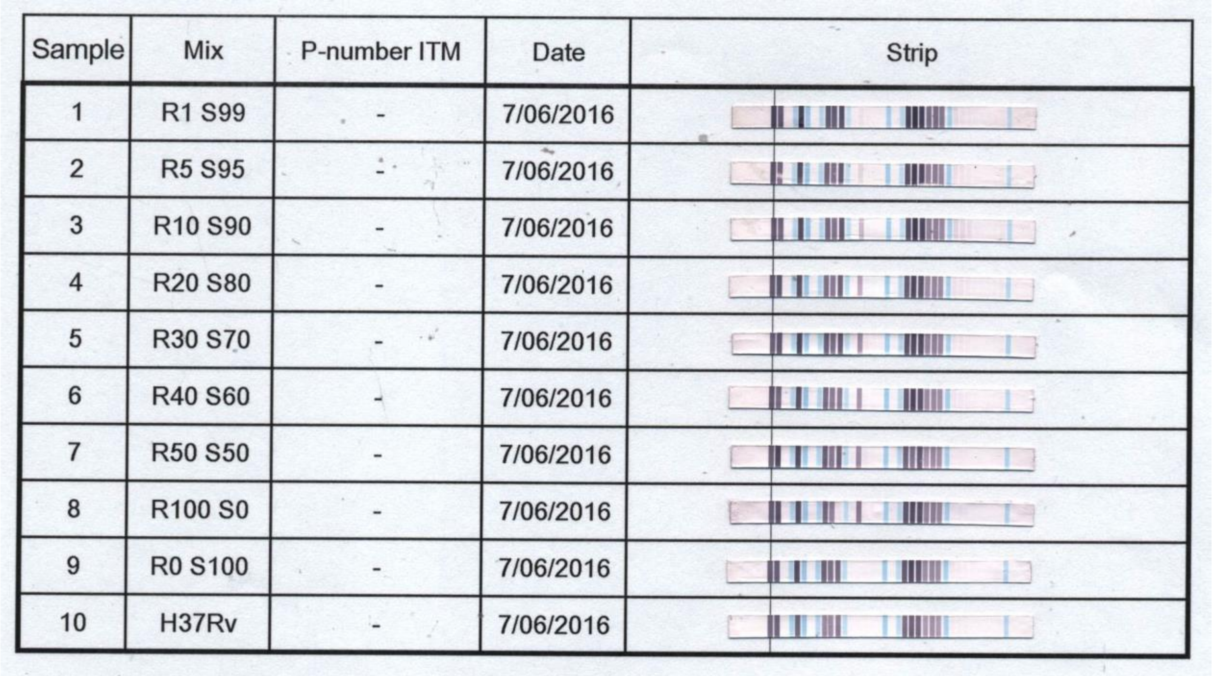

Sample	Mix	P-number ITM	Date	Strip
1	R1 S99	-	7/06/2016	
2	R5 S95	-	7/06/2016	
3	R10 S90	-	7/06/2016	
4	R20 S80	-	7/06/2016	
5	R30 S70	-	7/06/2016	
6	R40 S60	-	7/06/2016	
7	R50 S50	-	7/06/2016	
8	R100 S0	-	7/06/2016	
9	R0 S100	-	7/06/2016	
10	H37Rv	-	7/06/2016	

Supplementary Figure 4. Chromatograms (Finch TV Version 1.4.0, Geospiza Inc.) and alignments (CLC Sequence Viewer, Version 7.5; QIAGEN Aarthus A/S; reverse reading) from Sanger sequences for triplicates from experimentally mixed isogenic *Mycobacterium tuberculosis* strains in the following proportions (R%S%): R1S99, R5S95, R10S90, R20S80, R30S70, R40S60, R50S50, R100S0, R0S100, and the H37Rv reference strain (lanes A to J respectively) A double peak indicates heteroresistance.

More detailed information on the mutant proportion as determined by digital PCR can be found in Table 1 of the manuscript.

Replicate 1

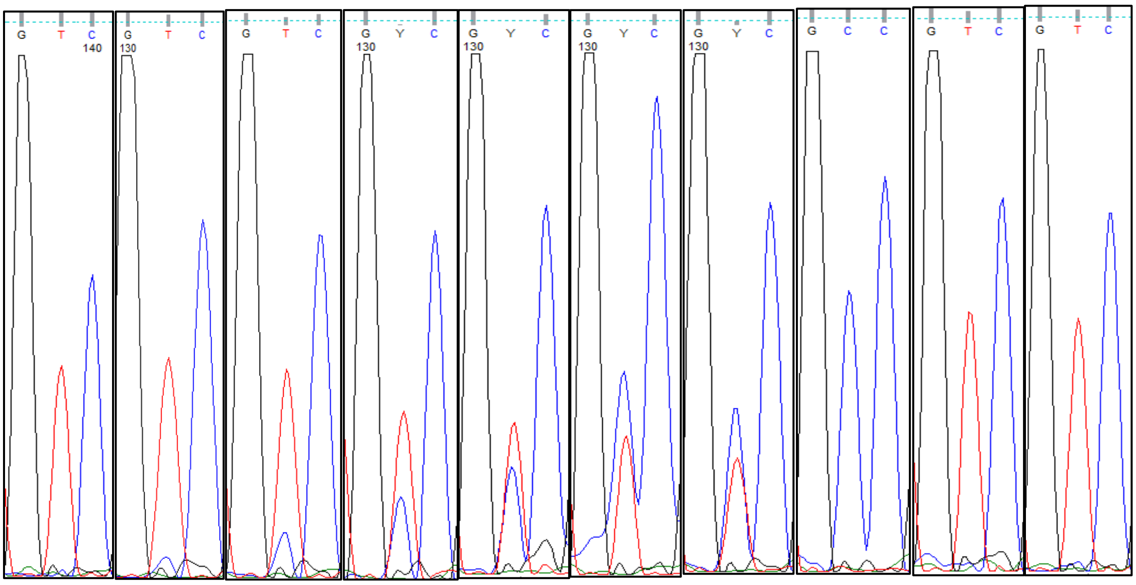

A	B	C	D	E	F	G	H	I	J

```
                                     2.320                                             2.340                                             2.360
                                       |                                                 |                                                 |
         H37Rv NC_000962.3 GGG CTG GGC CAT GCG CAC CAG GCT GTG TAG ATC GAC GCG TCG CCG TGC
p143254_GyraseAB-seq (A) ... ... ... ... ... ... ... .G. ... ... ... ... ... ... ... ...
p143255_GyraseAB-seq (B) ... ... ... ... ... ... ... .G. ... ... ... ... ... ... ... ...
p143256_GyraseAB-seq (C) ... ... ... ... ... ... ... .G. ... ... ... ... ... ... ... ...
p143257_GyraseAB-seq (D) ... ... ... ... ... ... ... .G. .Y. ... ... ... ... ... ... ...
p143258_GyraseAB-seq (E) ... ... ... ... ... ... ... .G. .Y. ... ... ... ... ... ... ...
p143259_GyraseAB-seq (F) ... ... ... ... ... ... ... .G. .Y. ... ... ... ... ... ... ...
p143260_GyraseAB-seq (G) ... ... ... ... ... ... ... .G. .Y. ... ... ... ... ... ... ...
p143261_GyraseAB-seq (H) ... ... ... ... ... ... ... .G. .C. ... ... ... ... ... ... ...
p143262_GyraseAB-seq (I) ... ... ... ... ... ... ... .G. ... ... ... ... ... ... ... ...
p143263_GyraseAB-seq (J) ... ... ... ... ... ... ... ... ... ... ... ... ... ... ... ...
```

Replicate 2

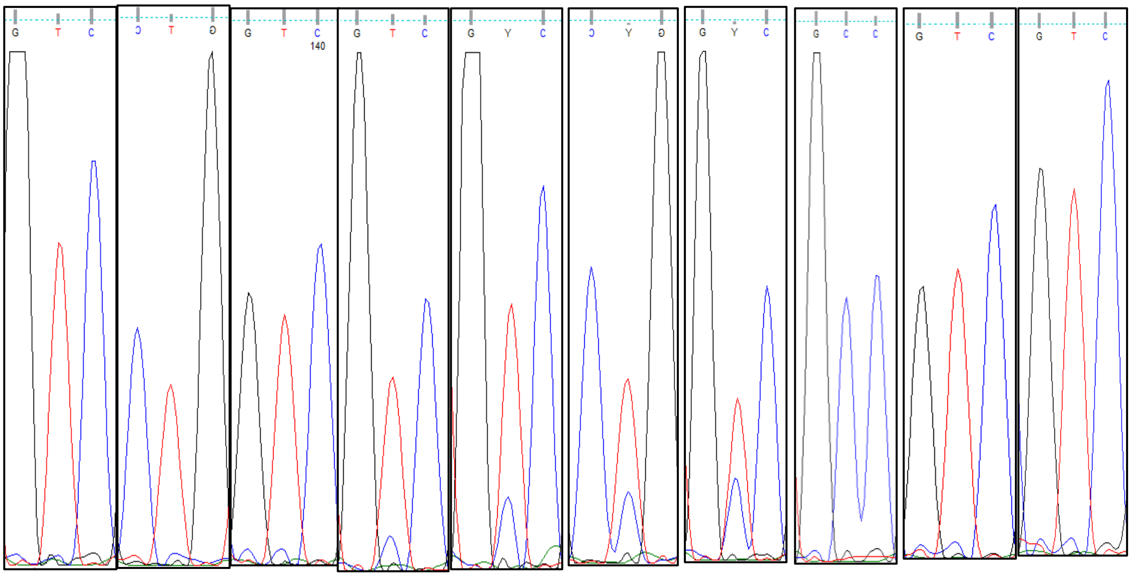

A	B	C	D	E	F	G	H	I	J

```
                                2.320                                                   2.340                                                  2.360
                                  |                                                       |                                                      |
      H37Rv NC_000962.3 GGG  CTG  GGC  CAT  GCG  CAC  CAG  GCT  GTC  GTA  GAT  CGA  CGC  GTC  GCC  GTG
p150130 GyraseAB-seq (A) ---  ---  ---  ---  ---  ---  ---  ---  ---  ---  ---  ---  ---  ---  ---  ---
p150131_GyraseAB-seq (B) ...  ...  ...  ...  ...  ...  ...  .G.  ...  ...  ...  ...  ...  ...  ...  ...
p150132_GyraseAB-seq (C) ...  ...  ...  ...  ...  ...  ...  .G.  .Y.  ...  ...  ...  ...  ...  ...  ...
p150133_GyraseAB-seq (D) ...  ...  ...  ...  ...  ...  ...  .G.  ...  ...  ...  ...  ...  ...  ...  ...
p150134_GyraseAB-seq (E) ...  ...  ...  ...  ...  ...  ...  .G.  .Y.  ...  ...  ...  ...  ...  ...  ...
p150135_GyraseAB-seq (F) ...  ...  ...  ...  ...  ...  ...  .G.  .Y.  ...  ...  ...  ...  ...  ...  ...
p150136_GyraseAB-seq (G) ...  ...  ...  ...  ...  ...  ...  .G.  .Y.  ...  ...  ...  ...  ...  ...  ...
p150137_GyraseAB-seq (H) ...  ...  ...  ...  ...  ...  ...  .G.  .C.  ...  ...  ...  ...  ...  ...  ...
p150138_GyraseAB-seq (I) ...  ...  ...  ...  ...  ...  ...  .G.  ...  ...  ...  ...  ...  ...  ...  ...
p150139_GyraseAB-seq (J) ...  ...  ...  ...  ...  ...  ...  ...  ...  ...  ...  ...  ...  ...  ...  ...
```

Replicate 3

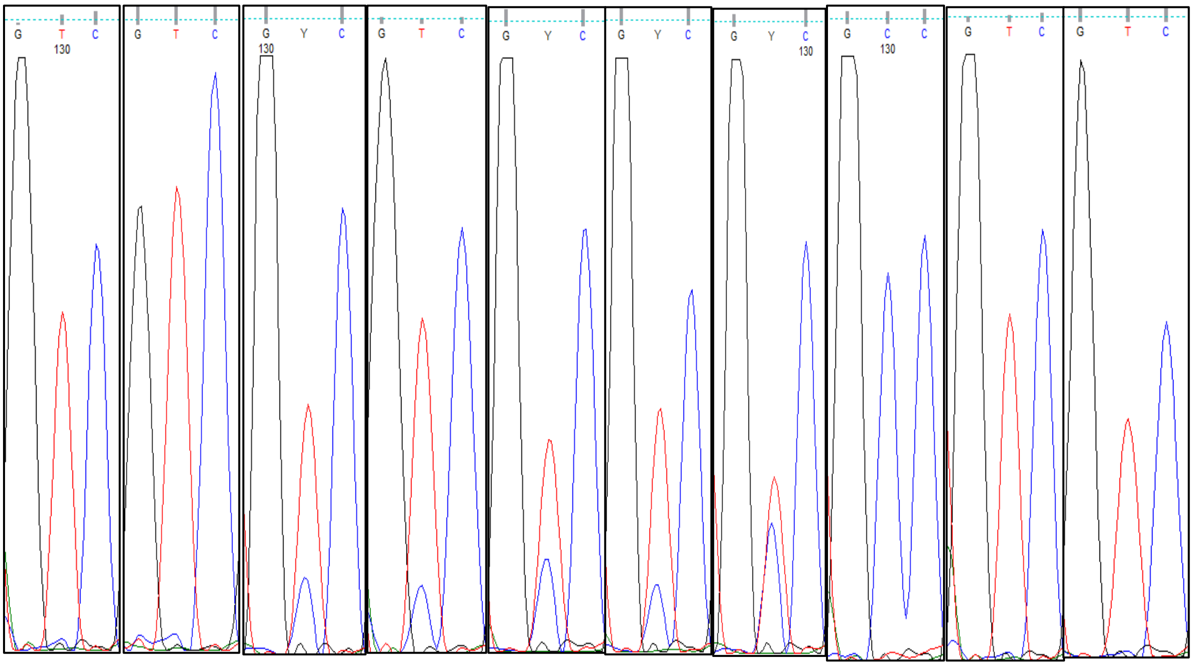

A	B	C	D	E	F	G	H	I	J

```
                                      2.320                                                    2.340                                                   2.360
                                        |                                                        |                                                       |
       H37Rv NC_000962.3 GGG  CTG  GGC  CAT  GCG  CAC  CAG  GCT  GTC  GTA  GAT  CGA  CGC  GTC  GCC  GTG
p150015_GyraseAB-seq (A) ...  ...  ...  ...  ...  ...  ...  .G.  ...  ...  ...  ...  ...  ...  ...  ...
p150016_GyraseAB-seq (B) ...  ...  ...  ...  ...  ...  ...  .G.  ...  ...  ...  ...  ...  ...  ...  ...
p150017_GyraseAB-seq (C) ...  ...  ...  ...  ...  ...  ...  .G.  ...  ...  ...  ...  ...  ...  ...  ...
p150018_GyraseAB-seq (D) ...  ...  ...  ...  ...  ...  ...  .G.  ...  ...  ...  ...  ...  ...  ...  ...
p150019_GyraseAB-seq (E) ...  ...  ...  ...  ...  ...  ...  .G.  .Y.  ...  ...  ...  ...  ...  ...  ...
p150020_GyraseAB-seq (F) ...  ...  ...  ...  ...  ...  ...  .G.  .Y.  ...  ...  ...  ...  ...  ...  ...
p150021_GyraseAB-seq (G) ...  ...  ...  ...  ...  ...  ...  .G.  .Y.  ...  ...  ...  ...  ...  ...  ...
p150022_GyraseAB-seq (H) ...  ...  ...  ...  ...  ...  ...  .G.  .C.  ...  ...  ...  ...  ...  ...  ...
p150023_GyraseAB-seq (I) ...  ...  ...  ...  ...  ...  ...  .G.  ...  ...  ...  ...  ...  ...  ...  ...
p150024_GyraseAB-seq (J) ...  ...  ...  ...  ...  ...  ...  ...  ...  ...  ...  ...  ...  ...  ...  ...
```

Supplementary Table: Results from the three replicates separately

	Replicate 1						
			LPA		Sanger sequence		
Suspen-sion	ddPCR (range %)	MIC (mg/L)	Genotype MTBDR*sl*	GenoScholar-FQ+KM TB II	Software calling	Manual inspection	Targeted NGS (%)
**R1**S99	**1.16 (0.93-1.39)**	0.5	WT	WT	A	A	1.82
**R5**S95	**9.2 (7.8-10.7)**	**1.0**	**WT+MUT3C**	**WT+R2c**[1]	A	A	9.62
**R10**S90	**14.4 (12.6-16.3)**	**1.0**	**WT+MUT3C**	**WT+R2c**[1]	**R**	**R**	17.96
**R20**S80	**28.3 (26-30.7)**	**1.0**	**WT+MUT3C**	**WT+R2c**[1]	**R**	**R**	30.56
**R30**S70	**35.1 (33.2-37)**	**1.0**	**WT+MUT3C**	**WT+R2c**[1]	**R**	**R**	39.64
R40**S60**	**45.1 (42.4-47.9)**	**1.0**	**WT+MUT3C**	**WT+R2c**	**R**	**R**	48.42
**R50**S50	**55.6 (53.3-57.8)**	**1.0**	**WT+MUT3C**	**WT+R2c**	**R**	**R**	57.67
**R100**S0	**100**	**1.0**	**MUT3C**	**R2c**	**G**	**G**	99.84
R0S100	0	<0.25	WT	WT	A	A	0.31
H37Rv	0	<0.25	WT	WT	A	A	0.23

	Replicate 2						
			LPA		Sanger sequence		
Suspen-sion	ddPCR (range %)	MIC (mg/L)	Genotype MTBDR*sl*	GenoScholar-FQ+KM TB II	Software calling	Manual inspection	Targeted NGS (%)
**R1**S99	0.06 (0-0.21)	0.5	WT	WT	A	A	0.41
**R5**S95	**3.7 (2.2-5.1)**	0.5	**WT+MUT3C**	WT	A	A	5.31
**R10**S90	**5.2 (4-6.4)**	**1.0**	**WT+MUT3C**	WT	A	A	6.40
**R20**S80	**11.7 (10.2-13.2)**	**1.0**	**WT+MUT3C**	WT	A	**R**	14.89
**R30**S70	**21.2 (18.7-23.7)**	**1.0**	**WT+MUT3C**	**WT+R2c**[1]	**R**	**R**	23.60
R40**S60**	**33.6 (30.9-36.4)**	**1.0**	**WT+MUT3C**	**WT+R2c**	**R**	**R**	34.17
**R50**S50	**38 (35.1-41)**	**1.0**	**WT+MUT3C**	**WT+R2c**	**R**	**R**	38.77
**R100**S0	**99.77 (99.53-100)**	**1.0**	**MUT3C**	**R2c**	**G**	**G**	99.73
R0S100	0	<0.25	WT	WT	A	A	0.31
H37Rv	not tested	<0.25	WT	WT	A	A	0.35

	Replicate 3						
			LPA		Sanger sequence		
Suspen-sion	ddPCR (range %)	MIC (mg/L)	Genotype MTBDR*sl*	GenoScholar-FQ+KM TB II	Software calling	Manual inspection	Targeted NGS (%)
**R1**S99	0.57 (0.34-0.8)	0.5	WT	WT	A	A	1.17
**R5**S95	**9.2 (7.7-10.6)**	**1.0**	**WT+MUT3C**	WT	A	A	11.24
**R10**S90	**21.6 (19.9-23.3)**	**1.0**	**WT+MUT3C**	**WT+R2c**[1]	**R**	**R**	23.35
**R20**S80	**16.9 (15.2-18.6)**	**1.0**	**WT+MUT3C**	**WT+R2c**	A	**R**	21.73
**R30**S70	**26.5 (28.4-24.5)**	**1.0**	**WT+MUT3C**	**WT+R2c**	**R**	**R**	29.60
R40**S60**	**20.1 (18.6-21.7)**	**1.0**	**WT+MUT3C**	**WT+R2c**	**R**	**R**	21.91
**R50**S50	**39.7 (37.8-41.4)**	**1.0**	**WT+MUT3C**	**WT+R2c**	**R**	**R**	45.25
**R100**S0	**99.94 (99.81-100)**	**1.0**	**MUT3C**	**R2c**	**G**	**G**	99.79
R0S100	0	<0.25	WT	WT	A	A	0.35
H37Rv	not tested	<0.25	WT	WT	A	A	0.23
